# How to improve prevention of hospital infection after cardiac surgery?

**DOI:** 10.1186/1749-8090-8-S1-P158

**Published:** 2013-09-11

**Authors:** B Mihajlovic, Lj Markovic - Denic, A Redzek, L Velicki, S Nicin, M Fabri

**Affiliations:** 1 Cardiovascular Surgery, Institute of Cardiovascular Diseases Vojvodina, Serbia; 2 Epidemiology, Institute of Epidemiology Medical Faculty Belgrade, Belgrade, Serbia

## Background

Hospital (nosocomial) infections are associated with higher morbidity and are one of the leading cause of death after cardiac surgery.The increased hospital length of stay, large use of antimicrobial drugs and the need for isolation significantly contribute to higher costs, as well.The aim of the study: to identify independent risk factors significantly contributing to development of nosocomial infection after cardiac surgery.

## Methods

The prospective study included 1075 consecutive patients (717 male vs. 311 female), who underwent cardiac surgical procedures from January to December 2012, at our Clinic. In order to detect the possible risk factors, the 52 variables (20 patients-related factors, 16 cardiac-related factors and 16 operation-related factors) were analyzed.In all cases the diagnosis of infection was clinically and/or microbiologically confirmed.

## Results

Hospital infection occurred in 4.45%(47/1075) patients.Surgical site infection, bloodstream infection, pneumonia and enterocolitis were registered in 24, 10, 9 and 4 patient respectively.Multivariate logistic regression showed following independent risk factors significantly contributing to development of hospital infection: female gender: p=0.031;Odds ratio 1.990(1.065-3.719), ejection fraction: p=0.013;Odds ratio 0.968(0.943-0.993), insulin dependent diabetes mellitus : p=0.024;Odds ratio 2.351(1.119-4.939),simultaneous carotid and coronary surgery: p=0.001;Odds ratio 12.324(2.913-52.133), National Nosocomial Infections Surveillance System (NNIS) risk index: p=0.031;Odds ratio 1.729(1.062-2.815).

## Conclusion

In order to improve our hospital infection control programme and to prevent hospital infections after cardiac surgery , special attention has to be paid to patients with the following risk factors: female gender, low ejection fraction, insulin dependent diabetes mellitus, simultaneous carotid and coronary surgery and higher NNIS risk index.

